# Supervised representation learning based on various levels of pediatric radiographic views for transfer learning

**DOI:** 10.1038/s41598-024-58163-y

**Published:** 2024-03-30

**Authors:** Sunggu Kyung, Miso Jang, Seungju Park, Hee Mang Yoon, Gil-Sun Hong, Namkug Kim

**Affiliations:** 1 Department of Convergence Medicine, Asan Medical Center, University of Ulsan College of Medicine, 88 Olympic-Ro 43-Gil, Songpa-gu, Seoul, 05505 Republic of Korea; 2 Department of Radiology and Research Institute of Radiology, Asan Medical Center, University of Ulsan College of Medicine, 88 Olympic-Ro 43-Gil, Songpa-gu, Seoul, 05505 Republic of Korea; 3 Present Address: Department of Biomedical Engineering, Asan Medical Institute of Convergence Science and Technology, Asan Medical Center, College of Medicine, University of Ulsan, Seoul, Republic of Korea

**Keywords:** Pediatric radiographs, Representation learning, Transfer learning, Bone age assessment, Deep learning, Fracture classification, Computer science, Software, Biomedical engineering

## Abstract

Transfer learning plays a pivotal role in addressing the paucity of data, expediting training processes, and enhancing model performance. Nonetheless, the prevailing practice of transfer learning predominantly relies on pre-trained models designed for the natural image domain, which may not be well-suited for the medical image domain in grayscale. Recognizing the significance of leveraging transfer learning in medical research, we undertook the construction of class-balanced pediatric radiograph datasets collectively referred to as PedXnets, grounded in radiographic views using the pediatric radiographs collected over 24 years at Asan Medical Center. For PedXnets pre-training, approximately 70,000 X-ray images were utilized. Three different pre-training weights of PedXnet were constructed using Inception V3 for various radiation perspective classifications: Model-PedXnet-7C, Model-PedXnet-30C, and Model-PedXnet-68C. We validated the transferability and positive effects of transfer learning of PedXnets through pediatric downstream tasks including fracture classification and bone age assessment (BAA). The evaluation of transfer learning effects through classification and regression metrics showed superior performance of Model-PedXnets in quantitative assessments. Additionally, visual analyses confirmed that the Model-PedXnets were more focused on meaningful regions of interest.

## Introduction

The recent development of the convolution neural network (CNN)^1–4^ of deep learning has shown the human-level performance of computer vision in ImageNet large scale visual recognition challenge (ILSVRC)^5^. The number of studies showing outstanding research results by applying deep learning technology to medical imaging has increased significantly in recent years^6^. Deep learning technology is also applied to pediatric domains, solving various tasks or showing excellent research results such as disease classification^7,8^, segmentation^9^, bone age assessment (BAA)^10^, and device detection^11^. However, despite the impressive achievements of previous studies, there are still various obstacles to the real-world clinical application for pediatric tasks. In the realm of pediatric healthcare, the accessibility of AI-driven medical imaging solutions that exhibit dependable performance falls behind that of the adult population. This disparity can be attributed, at least in part, to the obstacles stemming from the stringent regulatory framework governing medical devices for use in children, as well as the limited availability of robust training data essential for the development of trustworthy AI models^12^. These mainly need transfer learning because of data shortage.

Transfer learning is the enhancement of learning in a downstream task through the transfer of knowledge from a related upstream task that has already been trained^13^, so it is primarily used to alleviate data shortage problems, accelerate convergence during training, and improve performance^14–16^. The problem is, existing prevalent transfer learning relied inevitably on the representation of the pre-trained model by the natural image dataset—referred to as ImageNet—on RGB color space^7,8,10^, it may not be suitable for medical images such as radiographs, computed tomography, and magnetic resonance images. The medical images typically have a higher resolution than the nature images and single-channel images in grayscale space. Thus, it could have a large domain shift when transferring^17^. According to the research^18^, in a large chest radiograph dataset task, ImageNet pre-trained model had a positive transfer effect on disease classification in most CNN models, but some models, including InceptionV3^4^, had a negative transfer effect. These results indicated we need to verify the effectiveness of ImageNet pre-trained models on various medical tasks. In addition, it is hard to interpret how the pre-trained models by natural images helped in the medical domain even if performance improves because the models could focus on minor local variations in texture. Due to the recent issue of ImageNet representation, there were recent studies in that the benefits of transfer learning with ImageNet may be limited that pre-training on in-domain medical imaging data is more effective^19,20^. However, it is difficult to collect medical images, especially pediatric radiographs for representation suitable for the medical domain, so research attempts are limited.

In this study, inspired by a class-balanced large-scale ImageNet dataset and its pre-trained representation, we first constructed the class-balanced pediatric radiographs datasets—referred to as PedXnets- by radiographic views labeling such as PedXnet-7C, PedXnet-30C, and PedXnet-68C. Subsequently, we conducted representation learning suitable for pediatric problems through a radiographic views’ classification task in a supervised manner. The Model-PedXnets, i.e. the pre-trained models on the PedXnets framework consists of upstream and downstream tasks; in an upstream task, we trained the network to learn the radiographic views information according to the range from seven basic classes to 68 detailed classes based on pediatric radiograph protocols; in a downstream task, transfer learning was conducted with the pre-trained models on PedXnets to solve the specific tasks including the fracture classification and the BAA. For verifying the effects of transfer learning with our radiographic views’ representations using PedXnets, it was compared with Model-Baseline, i.e., trained model with random initialization and without pre-trained representation, and Model-ImageNet, i.e., pre-trained model on ImageNet. In addition, an ablation study was performed to compare the effects of radiographic views representation in small-scale downstream datasets.

## Materials and methods

This retrospective study was conducted according to the principles of the Declaration of Helsinki and was performed in accordance with current scientific guidelines. The study protocol was approved by the Institutional Review Board Committee of Asan Medical Center, University of Ulsan College of Medicine, Seoul, Korea (IRB No. 2019-0115). The requirement of patient informed consent was waived by the Institutional Review Board Committee of Asan Medical Center.

### Upstream: pediatric dataset

**Figure 1:**
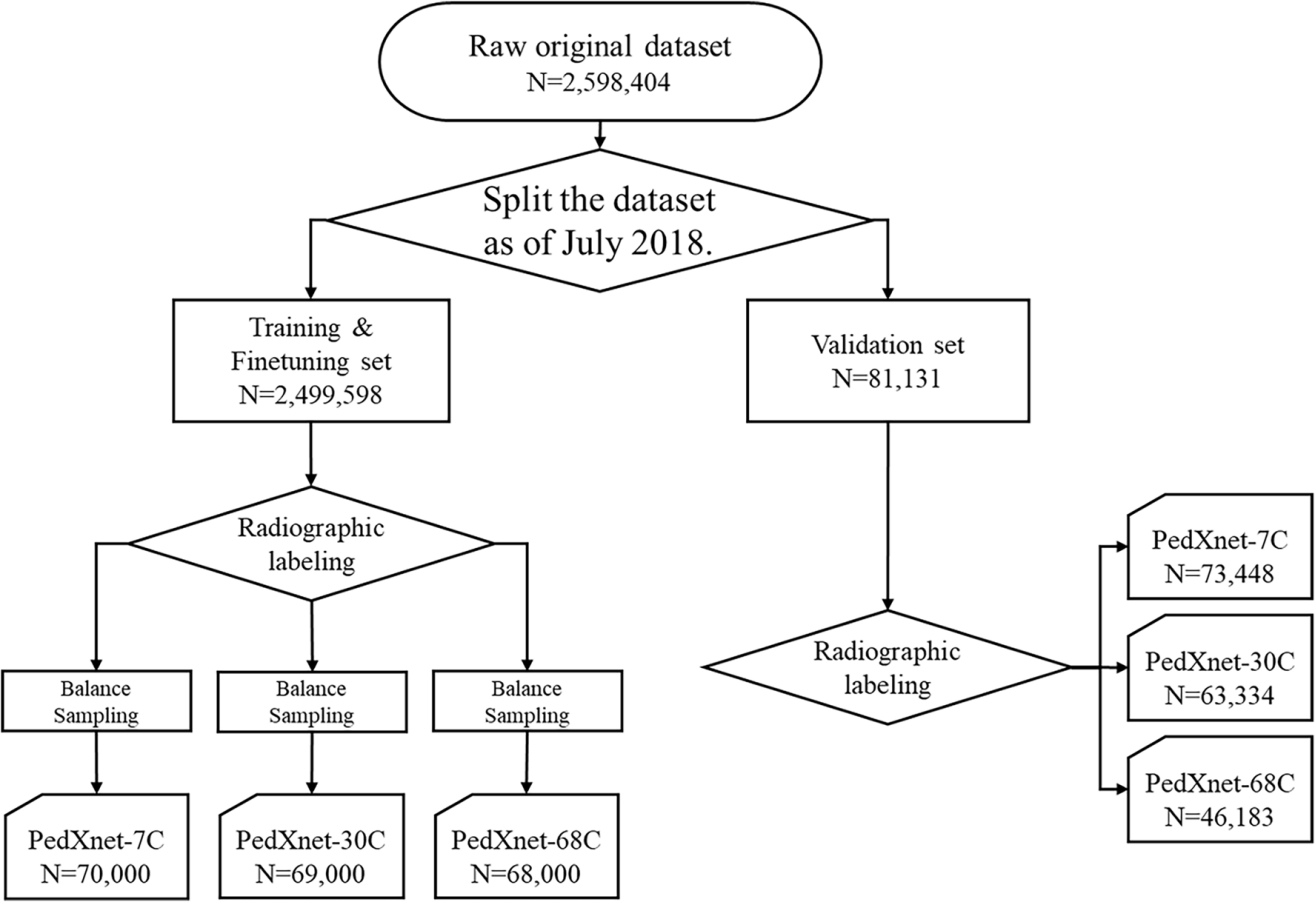
Flow chart of processing of upstream dataset with proposed radiographic views labeling in real-world medical radiographs dataset. Sampling was performed independently to build balanced datasets for each type of radiographic views labeling. In the case of the upstream validation set, the same radiographic views labeling was applied to the fixed dataset after a reference date (2018 Jul), so the class-wise mean and variance per labeling type were different. *N* the total number of the data.

A total of 2,598,404 pediatric radiographs were collected from 1995 to 2018 at Asan Medical Center (AMC) retrospectively, and we define this dataset raw original data (see Fig. 1). The age range of the original dataset was from 0 to 18. For reflection on the actual frequency of occurrence in the medical center, we divided the original dataset into the training and validation set based on the reference date; 2018 Jul. The validation set in the original dataset consists of a total of 81,131 radiographs over the period of 2018 Jul to 2018 Dec. The training dataset in the original dataset was composed of the remaining 2,499,598 radiographs. The original dataset had a severe imbalance distribution by prescription code. Therefore, when applying our proposed radiographic views labeling, the imbalance by class became highly severe. To address this, we under-sampled data according to the least frequent class and matched the total number equally for a fair comparison between PedXnet-7C, PedXnet-30C, and PedXnet-68C datasets. After the sampling, PedXnet-7C dataset consisted of 70,000 total (i.e., 10,000 radiographs per class), PedXnet-30C dataset consisted of 69,000 total (i.e., 2,300 radiographs per class), and PedXnet-68C dataset consisted of 68,000 total (i.e., 1,000 radiographs each class). Additionally, we constructed a fine-tuning set by separating 10% per class from each training set for hyper-parameter tuning. For more information on the class details of the upstream datasets by radiographic views labeling type (see Supplementary Tables 1–3). The baseline characteristics of upstream dataset are in the Supplementary Table 5.

### Downstream: fracture dataset

We utilized the publicly available GRAZPEDWRI-DX dataset^21^, comprising annotated pediatric trauma wrist radiographs from 6091 patients who received treatment in the Department of Pediatric Surgery at the University Hospital Graz between 2008 and 2018. This dataset contains 20,327 images, predominantly featuring posteroanterior and lateral views. It represents a wide range of patient demographics, with a mean age of 10.9 years (ranging from 0.2 to 19 years; comprising 2,688 females, 3402 males, and one individual of unknown gender). To create a binary classification dataset, we filtered the ‘fracture visible’ column from the annotations to differentiate between ‘fracture’ and ‘no fracture’ categories, thereby securing a binary label. The ratio of fractures to non-fractures was established at 2:1. The dataset was randomly divided into training, fine-tuning, and validation sets, adhering to a 3:1:1 ratio.

### Downstream: bone age prediction dataset

The dataset was released in RSNA Pediatric Bone Age Challenge (2017). The organizers provided the lists of training, fine-tuning, and validation sets. According to Halabi et al.^10^, the training and fine-tuning sets had similar age distributions with an average of 127.321 and 127.156 months, and the validation set had an age distribution with an average of 132.096 months. Radiographs for the training and fine-tuning sets were obtained from Children’s Hospital Colorado (Aurora, Colo) and Lucile Packard Children’s Hospital at Stanford. pediatric radiographs for the validation set were collected from Lucile Packard Children’s Hospital. The radiographs were provided with skeletal age estimates and sex from the accompanying clinical radiology report provided at the time of imaging. The Greulich and Pyle standard method (G-P method)^22^ was used by reviewers to determine the ground truth bone age.

### Preprocessing

For each image, two simple pre-processing methods were applied. First, min–max normalization with 0.5% clipping of upper and lower bounds was performed to suppress the effect of the L/R mark in radiographs and remove the outlier pixel values. We utilized the raw DICOM (Digital Imaging and Communications in Medicine) files as is, hence the min–max normalization was applied to the stored bit value range. A set of pixel values of original and scaled images is represented by X, Z respectively; the formula of min–max normalization is as follows:

1 $$Z= \frac{X-{\text{min}}(X)}{{\text{max}}\left(X\right)-{\text{min}}(X)}.$$

Second, due to the limitation of GPU resources, all images size were resized down into 512 × 512 by bi-cubic interpolation with keeping the aspect ratio. The size of the image is based on the Kim et al*.*^23^. Due to the characteristics of pediatric radiographs, there are various radiographic views protocols and the various size of the body depending on the age. Thus, we used strong image augmentations to alleviate the heterogeneity of the pediatric radiographs and make the model become robust t pediatric radiograph protocols in various anatomic locations. We used the image augmentation library, Albumentation^24^, and adopted the eight augmentation methods as follows: ShiftScaleRotate, HorizontalFlip, RandomBrightness, RandomContrast, RandomGamma, GaussNoise, Sharpen, and RandomBlur. Considering these previous studies^25,26^, we set the appropriate batch size emphatically depending on the upstream and downstream tasks. The batch sizes of upstream and downstream tasks were 60 and 20. Each model is initialized by a uniform Xavier and trained with an Adam optimizer, a learning rate of 1e−4 using a warm-up of 5 epochs, weight decay of 5e−4, and betas of (0.9, 0.999). The learning rate was reduced during the training following the polynomial learning rate schedule: ${(1-epoch{/epoch}_{max})}^{0.9}$.The total number of epochs is up to 500. However, each model was selected in the experiments as a converged model that has recorded the highest validation scores. All our models were implemented in Python version 3.6.9 with Pytorch version 1.6.0, accelerated by an NVIDIA TITAN RTX 24 GB graphics processing unit (GPU).

### Radiographic views labeling for PedXnets

**Figure 2:**
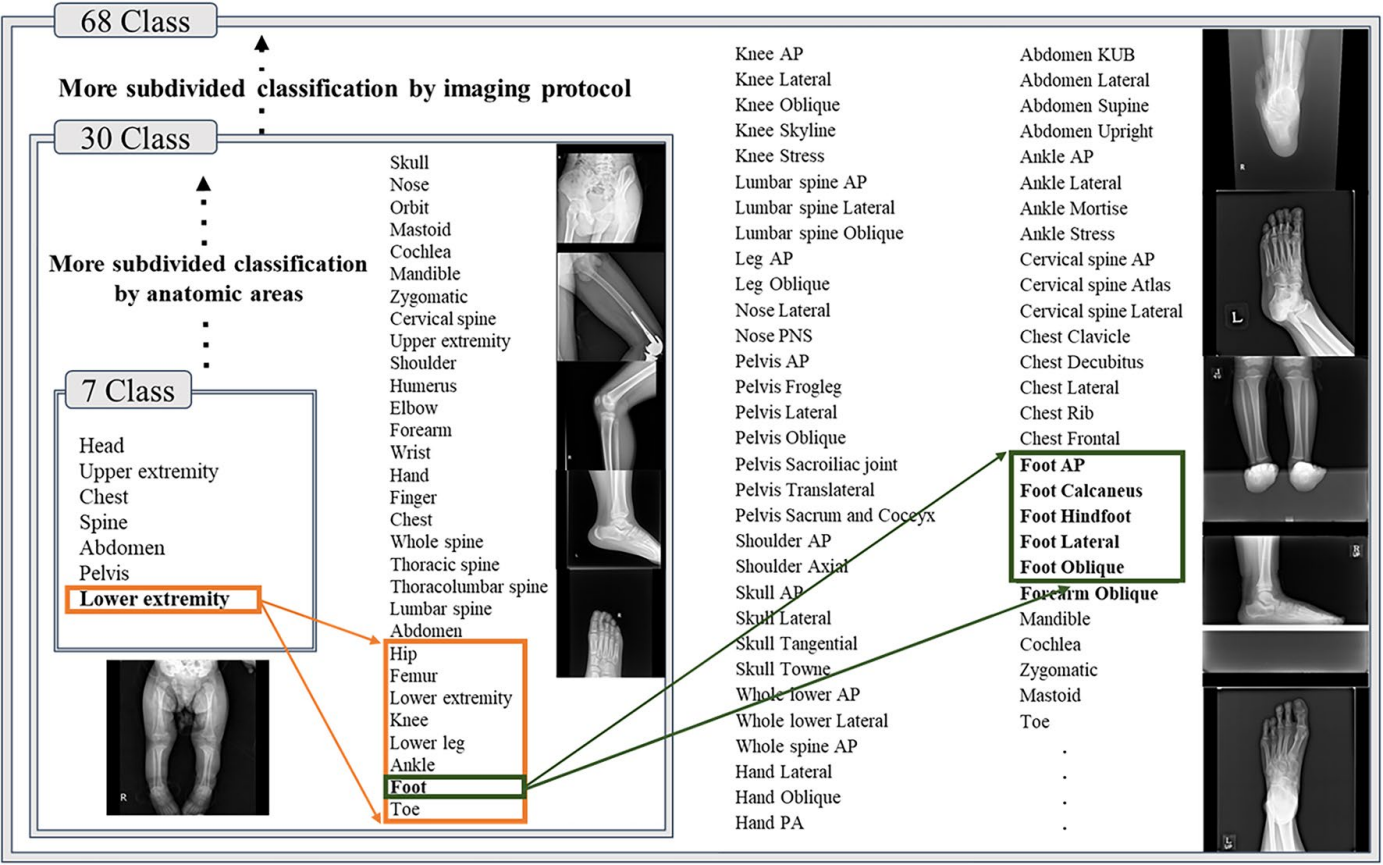
Overview of radiographic views labeling process for PedXnets. i.e., the lower extremity class in the 7 class can be divided into several classes: hip, femur, knee, lower leg, ankle, foot, and toe in the 30 class. These can be divided into several subclasses based on the protocol code of radiographs: Foot AP, Foot Calcaneus, Foot Hindfoot, Foot Lateral, and Foot Oblique in the 68 class. *PA* posteroanterior, *AP* anteroposterior, *KUB* kidney, ureter, and bladder, *PNS* paranasal sinus, *SI* sacroiliac.

We benchmarked the balanced class dataset, ImageNet^1^, and its hierarchical structure labeling based on WordNet^27^. As shown in Fig. 2, a hierarchical structure could be constructed with anatomical information for a major 7classes and radiographic views information for 68 classes in a large-scale original pediatric dataset. In detail, we divided it into seven major anatomic areas of the human body including the head, chest, upper extremity, abdomen, pelvis, spine, and lower extremity with all pediatric radiographs for construction of the PedXnet-7C. Furthermore, we subdivided from the 7 classes to 30 classes based on the detailed anatomic areas of radiographs for the composition of the PedXnet-30C; and much subdivided into 68 classes using radiographic protocols of radiographs for configuration of the PedXnet-68C. As shown in Fig. 1, there were strong imbalances between classes in the process of performing these radiographic views labeling in the collected raw original pediatric dataset. Thus, we built PedXnet-7C, PedXnet-30C, and PedXnet-68C using random sampling to construct balanced datasets among classes with the total number of training data.

### Supervised representation learning using radiographic views label

**Figure 3:**
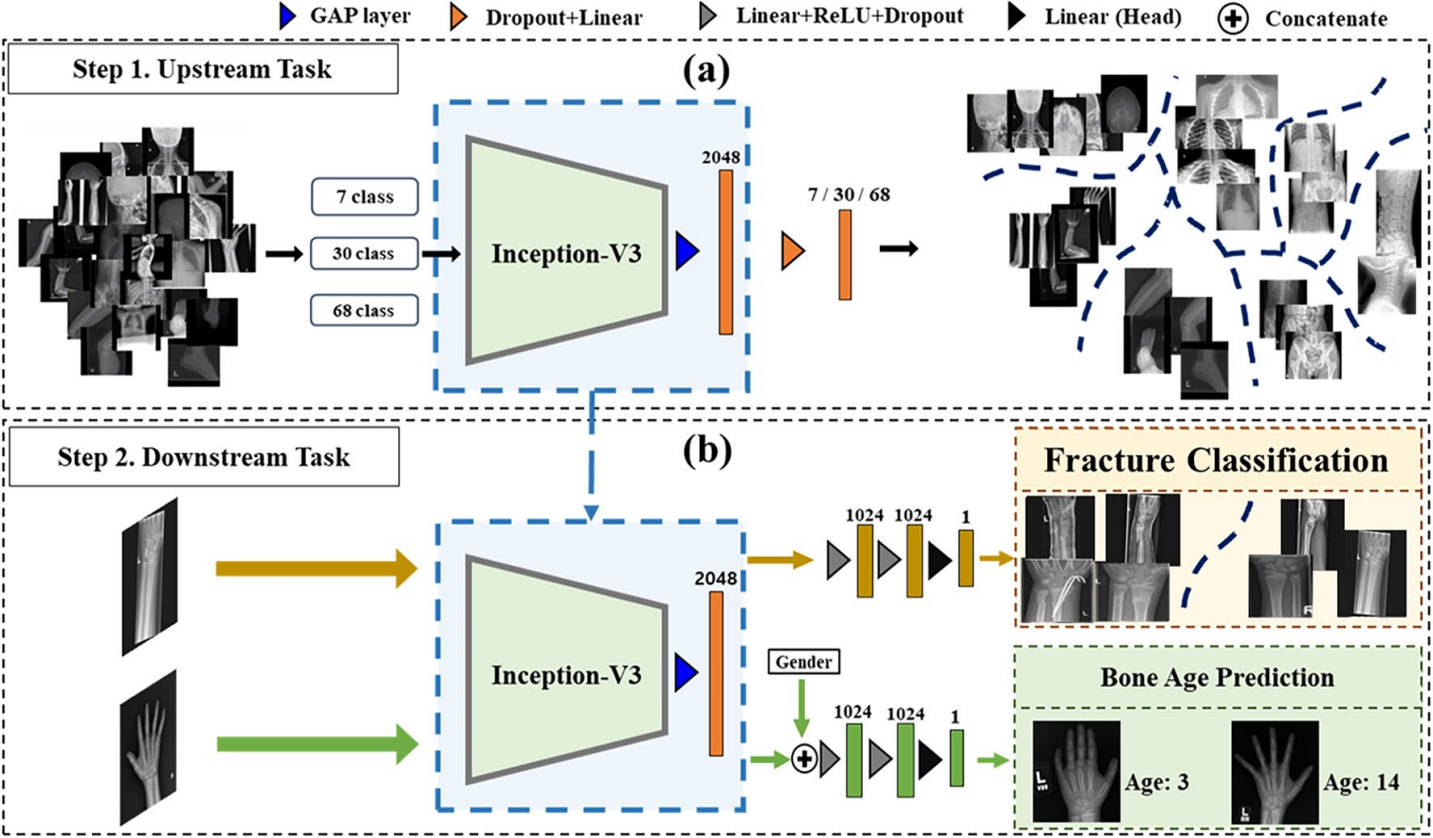
Overview of the Model-PedXnets framework. The framework consists of upstream and downstream tasks. In upstream tasks, radiographic views recognition of pediatric radiographs as a pretext for developing pre-trained models. In downstream tasks, transfer learning with the pre-trained weights for solving two medical problems including fracture classification and bone age assessment. *GAP* global average pooling, *ReLU* rectified linear unit.

We performed radiographic views recognition tasks as upstream tasks with our PedXnet-7C, PedXnet-30C, and PedXnet-68C for making the model capture the representation of radiographic views information of radiographs (see Fig. 3a). The models were trained to classify pediatric radiographs into each corresponding radiographic views class. The classification task loss was defined as the cross-entropy loss (CE loss), as follows:

2 $$Cross\, entropy\, loss\left(y,\widehat{y}\right)= -\frac{1}{N}\sum_{i=1}^{N}\sum_{c=1}^{M}{y}_{i}{\text{log}}\left(\widehat{{y}_{i}}\right),$$

where M is the number of classes, $\widehat{y}$ is the probability of M dimension outputs and $y$ is the M dimension one-hot encoded ground truth. For radiographic views classification tasks, InceptionV3^4^, a widely used CNN architecture since the ILSVRC 2015, was chosen, because InceptionV3 is recognized for its performance and is often used in medical problems e.g., detecting fractures^8,28^ and BAA^10^. In addition, according to Ke et al.^18^, when transfer learning was performed on 14 radiological observations classification tasks on chest radiographs using ImageNet pre-trained weight, the performance of InceptionV3 was rather lower than when ImageNet pre-trained weight was not used. Thus, the inceptionV3 was selected as a basic CNN architecture to find a suitable representation for the medical domain. The InceptionV3 has 11 convolution layers of 1 $\times$ 1, 1 $\times$ 3, and 1 $\times$ 5 kernels, and convolution blocks are applied along with the max-pooling layer for down sampling. All convolutional layers include batch normalization techniques and rectified linear unit (ReLU) layers. In the upstream tasks, predictions are conducted for target classes with fully connected layer and SoftMax function. We redesigned the last fully connected layer’s output dimension to the number of classes of each upstream task to perform the pre-defined tasks.

### Transfer learning for medical problems

To assess whether our proposed radiographic views representations by PedXnets benefit applications for medical problems, we conducted two pediatric downstream tasks; Fracture classification, BAA (see Fig. 3b). First, the classification of fractures in the upper and lower extremity of pediatric radiographs is considerably important^29,30^. A fracture can occur anatomically anywhere and frequently take place in childhood. In particular, fractures occur mainly in the upper and lower limbs of the body. Therefore, the model should be able to recognize fractures features robustly in multi-view of radiographs. The task could evaluate the transferability of Model-PedXnets at the multi-view task. Second, BAA in hand pediatric radiographs is also meaningful for evaluating the transferability of Model-PedXnets at the single-view task. For each downstream task, the Model-Baseline, Model-ImageNet, Model-PedXnet-7C, Model-PedXnet-30C, and Model-PedXnet-68C were applied with the same training settings.

To solve the fracture classification task using transfer learning, the models should extract general features of fracture in the upper and lower extremities of radiographs. We trained the Model-Baseline from scratch and conducted transfer learning using the Model-PedXnet-7C, Model-PedXnet-30C, Model-PedXnet-68C, and Model-ImageNet for the binary classification task of fractures in the upper and lower extremities of radiographs. The classification task loss is defined as the binary cross-entropy loss (BCE loss), as follows:

3 $$Binary \,Cross\, Entropy\, Loss\left(y,\widehat{y}\right)= -\frac{1}{N}\sum_{i=1}^{N}\left[{y}_{i}{\text{log}}\left(\widehat{{y}_{i}}\right)+\left(1-{y}_{i}\right){\text{log}}\left(1-\widehat{{y}_{i}}\right)\right],$$

where $\widehat{y}$ is the probability of model output and $y$ is the ground truth. The same preprocessing process and augmentations at the upstream task were performed but inspired by Parveen et al.^31^ but contrast limited adaptive histogram equalization (CLAHE)^32^ was additionally applied to emphasize the bone contrast. For a fair comparison, the same batch size, optimizer, learning rate, and scheduler at the upstream tasks were used except total epoch. The number of epochs at this task is up to 300. However, each model was selected at a converged model that has recorded the highest validation scores.

To solve the bone assessment task using transfer learning, BAA is mainly measured from hand radiographs, and the model should extract detailed features from the bones of the wrist, hand, and finger in only hand anteroposterior radiographs. We trained the Model-Baseline from scratch and executed transfer learning using the Model-PedXnet-7C, Model-PedXnet-30C, Model-PedXnet-68C, and Model-ImageNet for the regression task of bone age in the hand radiographs. The regression task loss is defined as the mean square error loss (MSE loss), as follows:

4 $$Mean \,Square\, Error\, Loss\left(y,\widehat{y}\right)= \frac{1}{N}\sum_{i=1}^{N}{[{y}_{i}-\widehat{{y}_{i}}]}^{2},$$

where $\widehat{y}$ is the probability of model output and $y$ is the ground truth. The same preprocessing process and augmentations at the upstream task were performed, but we additionally adopted the CLAHE to emphasize the bone contrast as in Halabi et al.^10^.

### Statistical analysis

To validate the Model-PedXnets representation in the upstream task, we employed F1-score (F1), accuracy (ACC), precision, and recall for the quantitative evaluations^33^ of multi-class classification in the upstream tasks. We visualized the Model-PedXnets representation with gradient-weighted class activation mapping (Grad-CAM)^34^ and t-distributed stochastic neighbor embedding (t-SNE)^35^ according to the anatomical hierarchy of radiographic views. To evaluate the effects of transfer learning with the pre-trained model on PedXnets in the downstream tasks, in the fracture task, receiver operating characteristic (ROC), the area under the ROC curve (AUC), F1, ACC, sensitivity (SEN), specificity (SPE), positive predictive values (PPV), and negative predictive values (NPV) were measured for binary-class classification, and in the BAA task, mean absolute error (MAE), mean square error (MSE), and R^2^ score were calculated for regression task. In addition, the activation maps were obtained using the Grad-CAM or channel-wise mean activation like Zhou et al.^36^ to interpret the representation of the Model-PedXnets in the downstream tasks. The output features of the last convolution layer of the InceptionV3 were used to be averaged channel-wise, normalized with sigmoid activation, and subsequently interpolated to match the input resolution. Each model was evaluated using weights saved at the minimum loss point of the fine-tuning set. The performance of each model was evaluated by the validation set. The comparisons of the Model-Baseline, Model-ImageNet, and Model-PedXnets in fracture classification and BAA were performed using DeLong’s ROC comparison^37^ and the paired t-test, respectively. The statistical significance level was set at the p-value of 0.05.

### Ethics approval

This retrospective study was conducted according to the principles of the Declaration of Helsinki and according to current scientific guidelines. The study protocol was approved by the Institutional Review Board Committee of Asan Medical Center, University of Ulsan College of Medicine, Seoul, Korea (IRB No.2019-0115).

### Consent to participate

The requirement of patient informed consent was waived by the Institutional Review Board Committee of Asan Medical Center.

## Results

### Upstream results of supervised radiographic views representation task

**Figure 4:**
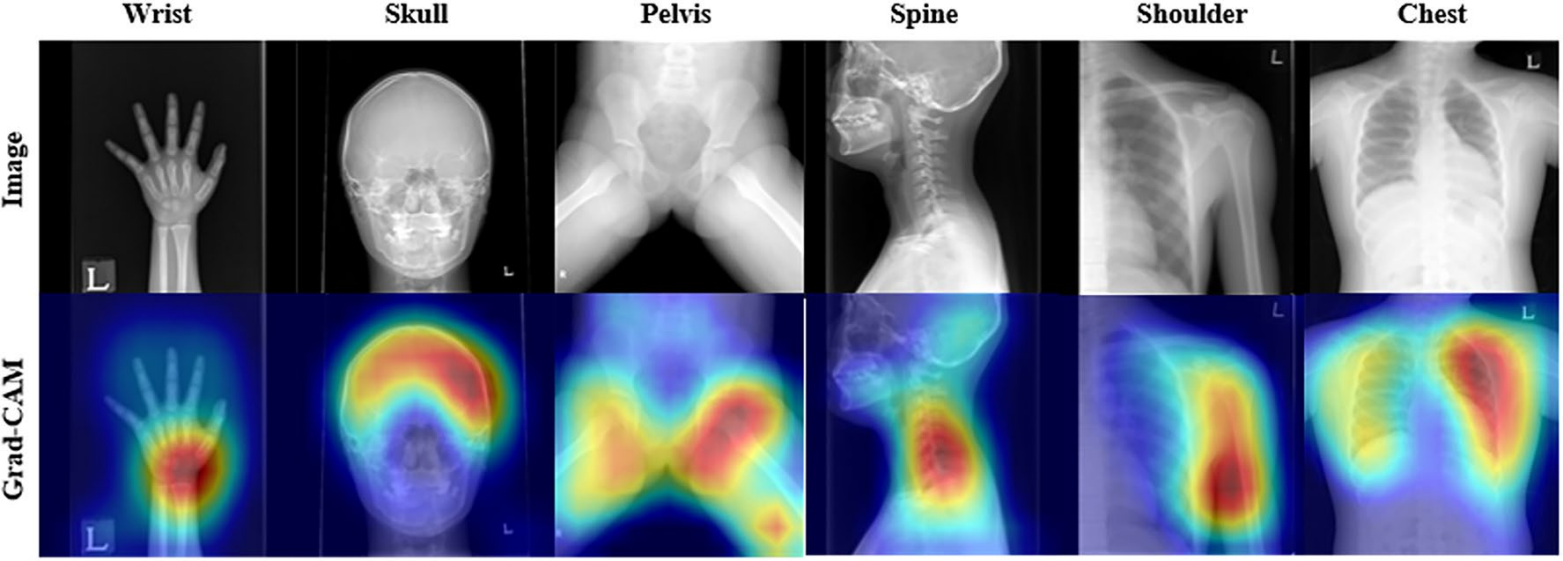
Plots of Model-PedXnet-7C model’s Grad-CAM activation maps of radiographic views recognition task as an upstream task with independent validation sets according to each labeling method. *Grad-CAM* gradient-weighted class activation mapping.

As shown in Table 1, when the highest value epoch model was selected from the fine-tuning set results and referred to the validation set, Model-PedXnet-7C, Model-PedXnet-30C, and Model-PedXnet-68C all have high performances (F1 > 0.78, Accuracy > 0.90, Precision > 0.84, Recall > 0.79). The upstream results indicated the Model-PedXnets learned the representation without overfitting, so we could use the model weights of the upstream task for application to downstream tasks. Figure 4 indicates Model-PedXnet-7C’s activation maps were visualized using Grad-CAM. The Model-PedXnet-7C was activated in the region of interest (ROI) and the activation maps demonstrate that Model-PedXnet-7C could capture clinically meaningful features. After pretraining, the Inception V3 model serves as a learnable feature extractor when applied to downstream tasks. It is initialized with its PedXnet pre-trained weights, excluding the last three layers that make up the fully connected layer. The training methodology for downstream tasks employs a comprehensive strategy that involves the entire model.

**Table 1:** The performance comparisons of radiographic views recognition task as an upstream task.

Upstream validation set	F1 score	Accuracy	Precision	Recall
Model-PedXnet-7C (N = 73,448)	0.892	0.911	0.915	0.874
Model-PedXnet-30C (N = 63,334)	0.823	0.952	0.933	0.797
Model-PedXnet-68C (N = 46,183)	0.785	0.904	0.847	0.798

### Downstream task result for fracture classification task

**Figure 5:**
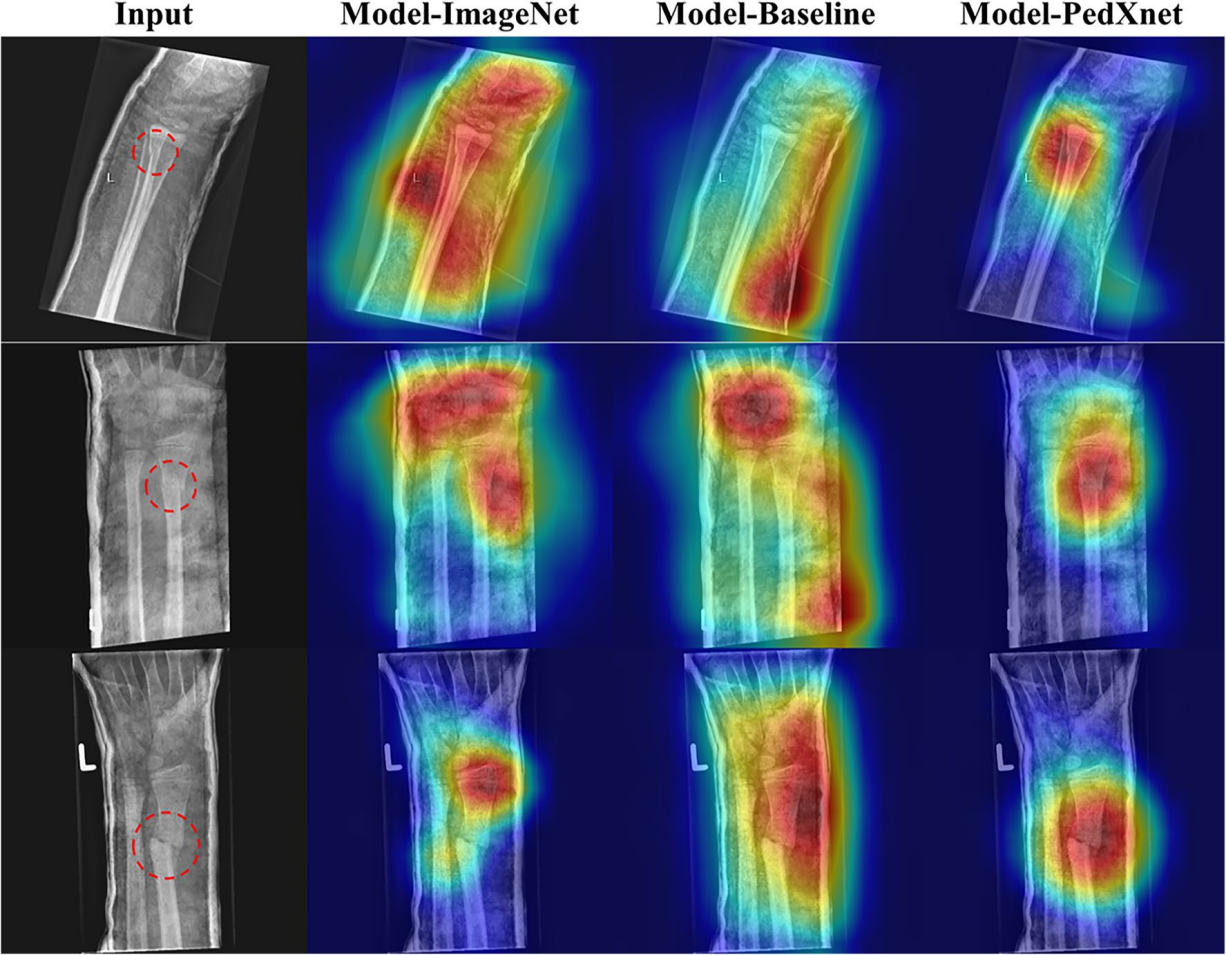
Comparisons of activation maps in the intermediate layer of Model-PedXnet, Model-Baseline, and Model-ImageNet models using Grad-CAM in the fracture downstream task. *Grad-CAM* gradient-weighted class activation mapping, *Model-Baseline* scratch model, *Model-PedXnet* the model pretrained from PedXnet-7class task, *Model-ImageNet* the model pretrained from ImageNet.

As presented in Table 2, a comparison between Model-PedXnets and Model-Baseline reveals that the AUC scores of Model-PedXnets significantly surpassed those of Model-Baseline, indicating statistically significant differences. Notably, Model-PedXnet-30C demonstrated superior performance across all metrics, with the exceptions of specificity, and PPV. The features of the last InceptionV3 convolution layer for Model-Baseline, Model-PedXnet-30C, and Model-ImageNet were visualized using Grad-CAM to verify their representations. The radiographs selected for this visualization were randomly chosen from the test dataset. As illustrated in Fig. 5, the Grad-CAM region of interest (ROI) for Model-PedXnet-30C is represented most effectively. Intriguingly, the Grad-CAMs for Model-Baseline demonstrate a biased focus towards casts, while that of Model-PedXnet shows the most concentrated depiction of a fracture lesion among the three models. We additionally validated the downstream fracture task using internal data from Asan Medical Center. For further details, please refer to the “Expansion of downstream task” section in the Supplementary Materials.

**Table 2:** The performance comparisons of the fracture classification task.

Network	AUC	F1 score	Accuracy	Sensitivity	Specificity	PPV	NPV
Model-baseline	0.912	0.874	0.838	0.829	0.857	0.925	0.701
Model-PedXnet-7C	0.969*	0.946	0.927	0.939	**0.901**	**0.953**	0.874
Model-PedXnet-30C	0.971*	**0.950**	**0.932**	**0.950**	0.891	0.949	**0.894**
Model-PedXnet-68C	0.966*	0.948	0.929	0.945	0.896	0.951	0.884
Model-ImageNet	0.967*	0.940	0.917	0.945	0.858	0.934	0.881

DeLong’s test method was adopted for pairwise ROC comparison between the baseline and each model.

Mean of all reader group is shown with 95% confidence interval.

*ROC* receiver operating characteristic, *AUC* area under the ROC curve, *PPV* positive predictive value, NPV Negative predictive value, *Model-Baseline* scratch model, *Model-PedXnet-7C* the model pretrained from PedXnet-7class task, *Model-PedXnet-30C* the model pretrained from PedXnet-30class task, *Model-PedXnet-68C* the model pretrained from PedXnet-68class task, *Model-ImageNet* the model pretrained from ImageNet.

*p < 0.05.

Significant values are in bold.

### Downstream task result for bone age assessment task

**Figure 6:**
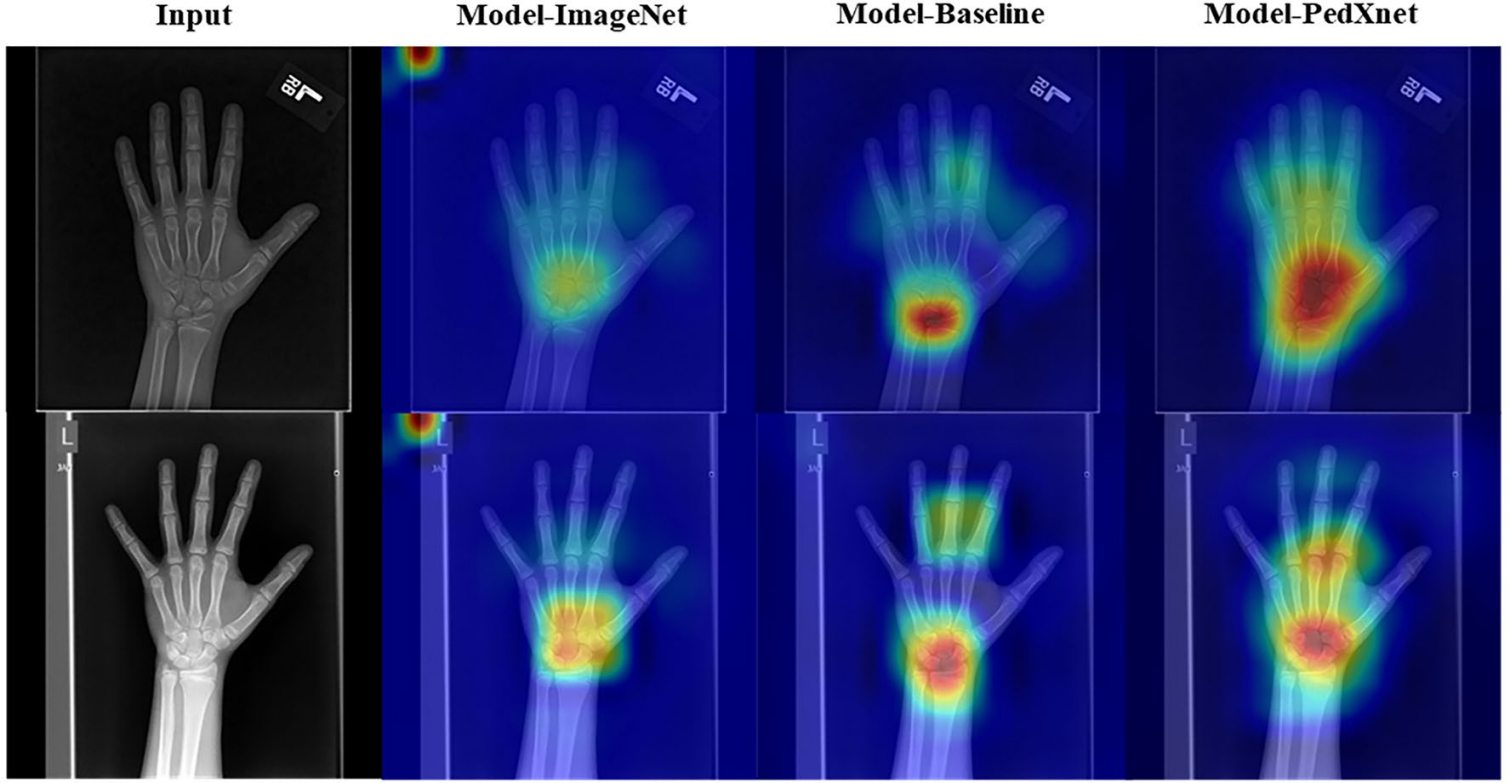
Plots of activation maps in the intermediate layer of Model-ImageNet, Model-Baseline, and Model-PedXnet using channel-wise mean activation map in the BAA. Please refer to Supplementary Fig. 4 for the activation map according to PedXnet types. The sample in the first row is 152 months old, and the sample in the second row is 167 months old. The carpus and metacarpophalangeal joints are critical regions for bone age assessment^39^. *BAA* bone age assessment, *Model-Baseline* scratch model, *Model-PedXnet* the model pretrained from PedXnet-7class task, *Model-ImageNet* the model pretrained from ImageNet.

Model-PedXnet-7C achieved the best performances of 5.245 in MSE, 42.857 in MAE, and 0.974 in R-square in the BAA task in Table 3. The Model-PedXnet-7C and Model-PedXnet-30C showed performance improvements in MAE compared to the baseline model. Figure 6 indicates that Model-PedXnet-7C captured the most important regions to predict bone age such as carpus and metacarpophalangeal joints, most intensively. The plotted radiographs were randomly chosen in the validation set. We added more plots of activation maps in Supplementary Fig. 3.

**Table 3:** The performances comparisons of bone age assessment.

Network	MAE (Month) ± Stdev.	MSE (month)	${R}^{2}$ score
Model-baseline	5.645 ± 4.576	52.694	0.968
Model-PedXnet-7C	**5.245 ± 3.927 (p-value: 0.241)**	**42.857**	**0.974**
Model-PedXnet-30C	5.567 ± 4.295 (p-value: 0.815)	49.347	0.971
Model-PedXnet-68C	5.851 ± 4.578 (p-value: 0.594)	55.082	0.970
Model-ImageNet	5.308 ± 4.422 (p-value: 0.213)	47.630	0.971

Paired t-test method was adopted for MAE comparison between the baseline and each model.

*stdev.* standard deviation, *MAE* mean average error, *MSE* mean squared error, *Model-Baseline* scratch model, *Model-PedXnet-7C* the model pretrained from PedXnet-7class task, *Model-PedXnet-30C* the model pretrained from PedXnet-30class task, *Model-PedXnet-68C* the model pretrained from PedXnet-68class task, *Model-ImageNet* the model pretrained from ImageNet.

Significant values are in bold.

## Discussion

Most of the previous medical tasks mainly use transfer learning because the scarcity of various cases and privacy protection issues cause the difficulty of medical data collection. Especially most pediatric studies rely on ImageNet representation. However, it is still debated whether the ImageNet representation is suitable for the medical domain^38^. In this study, we constructed the class-balanced pediatric dataset, PedXnets, and proposed our Model-PedXnets framework to reap the benefits of transfer learning in medical domains. In Tables 2 and 3, the Model-PedXnets showed superior performances improvements by a large margin compared to Model-Baseline in downstream pediatric tasks including fracture classification and bone age assessment. Even though using only approximately 70,000 images, PedXnets, smaller-scale datasets than ImageNet, the Model-PedXnets showed equal or superior performances compared with Model-ImageNet. The findings of this study revealed that data including medical content, even if it is not as large as ImageNet, is better for solving medical problems. To the best of our knowledge, this is the first study to demonstrate representative learning with pediatric radiographs and compare the effects of transfer learning with two major pediatric tasks.

In addition, these differences expressed in the activation maps were more pronounced in qualitative results. In the fracture downstream task, the Model-ImageNet focused on some minor local context, while the Model-PedXnet focused on more medically meaningful ROI. The Model-PedXnet accurately concentrates the fracture part without being affected by casts compared to other methods. In Supplementary Figs. 1 and 5, we presented radiographs of the upper and lower extremity and Model-PedXnet appropriately highlights the fracture site in various radiographic views. In the BAA downstream task, we presented some hand radiographs and activation maps in Supplementary Fig. 3 and Fig. 6. Our activation maps were dynamic changes according to age and important areas for predicting were carpus, thumb, and metacarpophalangeal joints^39^. Model-PedXnet less highlighted meaningless information with high intensity, such as L or R marks and lines of films in the radiographs. As shown in Supplementary Figs. 2 and 4, there was no significant difference in ROI activity between the Model-PedXnets in the downstream tasks. Because Model-PedXnets training strategy was designed to extract pediatric radiographs’ context features with the radiographic views labeling, which could help the models to understand the important pediatric regions of the radiographs.

As shown in Supplementary Table 4, similar results were shown in the ablation study where the number of training data was limited in the downstream task. In addition, comparing the results of the among Model-PedXnets, it was found that radiographic views representation made with fewer classes of datasets, Model-PedXnet-7C, was more effective, unlike ImageNet representation with 1000 various class distributions. Radiographs serve as essential tools for medical diagnosis, and due to the risks associated with radiation exposure, the protocols for their use are meticulously regulated. Especially, pediatric radiographs include views of various sizes according to age. Therefore, the excessive dividing of the data class up to the protocol of the radiographs could collect simple and almost identical images, which would decrease the transfer learning effects because it was a highly trivial task. It also occurred in overlapping regions between classes, for example, chest AP view images were similar to abdomen AP views in newborns and infants, which would act as a kind of label noise. The network would miss meaningful features and result in a negative transfer phenomenon. Additionally, the performance decline in more detailed classes may be due to the diminished training data per class as their number increases, likely leading to insufficient learning for the radiologic view in PedXnet-68C.

Despite the improved performance, our method has some limitations. First, as our proposed methodology can rely on the backbone network and pre-processing, it can lead to sufficiently different results by the different backbone networks and pre-processing. However, we fixed the InceptionV3 and the preprocessing because of the limited GPU in our study and left it open for discussion. Second, as shown in Fig. 1, we performed excessive random under-sampling in the raw original dataset to build class-balanced datasets according to the anatomical hierarchy of radiographic views. This has reduced the total number of training data and there might be a possibility that the total number of data was insufficient compared to ImageNet, so it did not show an appropriate effect^40^. Third, since the radiographic views labeling may vary depending on the radiologist and our proposed method is a supervised manner, the results could be greatly changed by the label method. Labeling the data class from an anatomical or radiographic perspective can be somewhat subjective. Fourth, the developed pretraining model was trained and validated exclusively on pediatric data. The domain gap between upstream and downstream tasks in pre-training research is a critical factor, as it can significantly impact the effectiveness of pretraining models. The influence of pretraining with pediatric data on other medical datasets remains an area for future investigation. Our research is focused on demonstrating the effects of pre-training through supervised learning, using the radiographic views labeling in pediatric data. Comparing the pretraining model with the adult chest X-ray dataset, CheXNet^41^, is considered as future work. Recent advancements in unsupervised learning and the growing need for pre-trained models tailored to medical domains have led to significant developments. Specifically, previous studies^42–44^ have successfully developed pre-trained weights designed for reconstruction tasks, resulting in substantial performance improvements in dense prediction tasks. Building on these foundations, future work will aim to establish a more effective framework for unsupervised representation of radiographic views. Fifth, we verified the effect of pretraining exclusively through the full fine-tuning method when applied to downstream tasks. Investigating the pretraining effect via other transfer learning techniques, such as Linear Probing and Gradual Unfreezing, remains a subject for future research. Sixth, to improve the reliability and objective quantification of the Grad-CAM results, we intend to incorporate the implementation of a blind test into future work. In this test, human evaluators will identify the most accurate Grad-CAM focus among images without knowledge of the model that produced each.

## Conclusion

In this study, we introduced a supervised manner of medical representation learning for pediatric tasks with radiographic views labels. First, we designed the class-balanced pediatric radiographs datasets (PedXnets) by radiographic views labelings. And by using the PedXnets, we conducted representation learning helpful for pediatric problems through a radiographic views’ classification task in a supervised manner. According to the evaluation results, the representation of major anatomical information was effective and the transfer effect of Model-PedXnet was positive in both pediatric downstream tasks including fracture classification and bone age assessment tasks. The Model-PedXnets showed superior results by a large margin compared to Model-Baseline and even showed results equivalent or improved to the Model-ImageNet even though the PedXnets were smaller than ImageNet. In addition, the proposed representation learning allowed networks to capture more semantic features in the ROI of radiographs. Our study could be helpful for medical domains, particularly pediatric radiographs research, which is difficult to collect data, so we aim to disclose the PedXnet’s weights.

### Supplementary Information

Supplementary Information.

## Data Availability

Data available upon request to corresponding author with allowance of IRB. After organizing the code, the code will be uploaded at https://github.com/babbu3682/PedXnet.
