# Supplementary material for: Supervised representation learning based on various levels of pediatric radiographic views for transfer learning
Source: Sci Rep. 2024 Mar 30;14:7551. doi: 10.1038/s41598-024-58163-y (PMC10981659; doi:10.1038/s41598-024-58163-y)
Supplement: Supplementary file 1 — Supplementary Information. [file 41598_2024_58163_MOESM1_ESM.docx]

**Supplementary Materials**

**1. Class details of upstream 3 PedXnets validation datasets**

Although the class balance was adjusted through random sampling in the upstream training dataset, in order to reflect the real-world distribution, there were class imbalances in the upstream validation datasets because it is divided into a specific date. **Tables 1**, **2**, and **3** represent detailed classes and distributions in the PedXnets.

| **PedXnet-7C dataset** | | |
| --- | --- | --- |
| **Class** | **Validation data (%)** | |
| Chest | 29,476 | (40.13 %) |
| Upper extremity | 13,423 | (18.28 %) |
| Lower extremity | 12,142 | (16.53 %) |
| Abdomen | 6,008 | (8.18 %) |
| Spine | 5,553 | (7.56 %) |
| Head | 5,537 | (7.54 %) |
| Pelvis | 1,309 | (1.78 %) |
| Total | 73,448 | |
| Mean | 10492.57 | |
| Stdev | 9354.90 | |

**Supplemental Table 1.** Summary of distributions of the PedXnet-7C dataset in upstream task.

| **PedXnet-30C dataset** | | | | | |
| --- | --- | --- | --- | --- | --- |
| **Class** | **Validation data (%)** | | **Class** | **Validation data (%)** | |
| Skull | 2,312 | (3.65 %) | Finger | 834 | (1.32 %) |
| Nose | 2,552 | (4.03 %) | Chest | 23,362 | (36.89 %) |
| Orbit | 3 | (0.00 %) | Whole spine | 3,372 | (5.32 %) |
| Mastoid | 107 | (0.17 %) | Thoracic spine | 192 | (0.30 %) |
| Cochlea | 14 | (0.02 %) | Thoracolumbar spine | 258 | (0.41 %) |
| Mandible | 39 | (0.06 %) | Lumbar spine | 398 | (0.63 %) |
| Zygomatic | 10 | (0.02 %) | Abdomen | 5,508 | (8.70 %) |
| Cervical spine | 1,333 | (2.10 %) | Lower leg | 1,058 | (1.67 %) |
| Upper extremity | 934 | (1.47 %) | Hip | 1,309 | (2.07 %) |
| Shoulder | 500 | (0.79 %) | Femur | 544 | (0.86 %) |
| Humerus | 673 | (1.06 %) | Lower extremity | 2,290 | (3.62 %) |
| Elbow | 3,439 | (5.43 %) | Knee | 1,824 | (2.88 %) |
| Forearm | 1,333 | (2.10 %) | Foot | 3,030 | (4.78 %) |
| Wrist | 1,512 | (2.39 %) | Ankle | 1,729 | (2.73 %) |
| Hand | 2,554 | (4.03 %) | Toe | 311 | (0.49 %) |
| Total | | | 63,334 | | |
| Mean | | | 2448.27 | | |
| Standard deviation | | | 5321.34 | | |

**Supplemental Table 2.** Summary of distributions of the PedXnet-30C dataset in upstream task.

| **PedXnet-68C dataset** | | | | | |
| --- | --- | --- | --- | --- | --- |
| **Class** | **Validation data (%)** | | **Class** | **Validation data (%)** | |
| Skull anteroposterior | 59 | (0.09 %) | Lumbar spine anteroposterior | 134 | (0.21 %) |
| Skull towne | 39 | (0.06 %) | Lumbar spine lateral | 64 | (0.10 %) |
| Skull tangential | 83 | (0.13 %) | Lumbar spine oblique | 18 | (0.03 %) |
| Skull lateral | 59 | (0.09 %) | Abdomen kidney, ureter and, bladder | 154 | (0.24 %) |
| Nose para nasal sinus | 2,850 | (4.50 %) | Abdomen supine | 1,769 | (2.79 %) |
| Nose lateral | 202 | (0.32 %) | Abdomen lateral | 262 | (0.41 %) |
| Orbit | 3 | (0.00 %) | Abdomen upright | 2,258 | (3.57 %) |
| Mastoid | 107 | (0.17 %) | Pelvis anteroposterior | 649 | (1.02 %) |
| Cochlea | 14 | (0.02 %) | Pelvis lateral | 17 | (0.03 %) |
| Mandible | 39 | (0.06 %) | Pelvis sacrum and coccyx | 21 | (0.03 %) |
| Zygomatic | 10 | (0.02 %) | Pelvis frogleg | 176 | (0.28 %) |
| Cervical spine anteroposterior | 3 | (0.00 %) | Pelvis sacroiliac joint | 9 | (0.01 %) |
| Cervical spine lateral | 123 | (0.19 %) | Pelvis oblique | 142 | (0.22 %) |
| Cervical spine atlas | 42 | (0.07 %) | Pelvis translateral | 49 | (0.08 %) |
| Upper extremity | 934 | (1.47 %) | Femur anteroposterior | 126 | (0.20 %) |
| Shoulder anteroposterior | 137 | (0.22 %) | Whole lower anteroposterior | 184 | (0.29 %) |
| Shoulder axial | 101 | (0.16 %) | Whole lower lateral | 1,637 | (2.58 %) |
| Humerus oblique | 154 | (0.24 %) | Knee anteroposterior | 24 | (0.04 %) |
| Elbow lateral | 410 | (0.65 %) | Knee lateral | 61 | (0.10 %) |
| Forearm oblique | 438 | (0.69 %) | Knee oblique | 493 | (0.78 %) |
| Wrist anteroposterior | 39 | (0.06 %) | Knee stress | 70 | (0.11 %) |
| Wrist oblique | 610 | (0.96 %) | Knee skyline | 219 | (0.35 %) |
| Hand posteroanterior | 2,769 | (4.37 %) | Leg anteroposterior | 1 | (0.00 %) |
| Hand lateral | 76 | (0.12 %) | Leg oblique | 308 | (0.49 %) |
| Hand oblique | 28 | (0.04 %) | Ankle anteroposterior | 47 | (0.07 %) |
| Finger | 834 | (1.32 %) | Ankle lateral | 49 | (0.08 %) |
| Chest rib | 96 | (0.15 %) | Ankle mortise | 305 | (0.48 %) |
| Chest frontal | 22,919 | (36.19 %) | Ankle stress | 26 | (0.04 %) |
| Chest lateral | 102 | (0.16 %) | Foot anteroposterior | 429 | (0.68 %) |
| Chest clavicle | 300 | (0.47 %) | Foot lateral | 899 | (1.42 %) |
| Chest decubitus | 229 | (0.36 %) | Foot oblique | 575 | (0.91 %) |
| Whole spine anteroposterior | 242 | (0.38 %) | Foot calcaneus | 91 | (0.14 %) |
| Thoracic spine anteroposterior | 145 | (0.23 %) | Foot hindfoot | 152 | (0.24 %) |
| Thoracolumbar spine | 258 | (0.41 %) | Toe | 311 | (0.49 %) |
| Total | | | 46,183 | | |
| Mean | | | 679.16 | | |
| Stdev | | | 2801.79 | | |

**Supplemental Table 3.** Summary of distributions of the PedXnet-68C dataset in upstream task.

**2. Grad-CAM figures of fracture task**

**
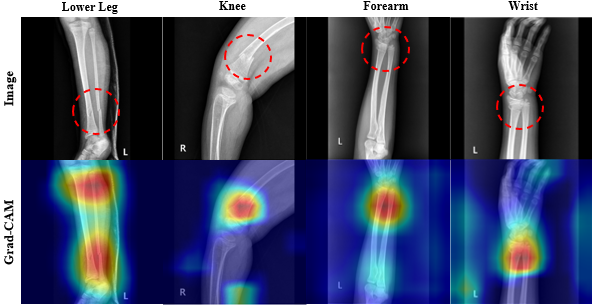
**

**Supplemental Figure 1.** The plots of Grad-CAM of Model-PedXnet-7C in the AMC fracture classification task. The left two figures are the case for the lower extremity class and the right two figures are the case for the upper extremity class.


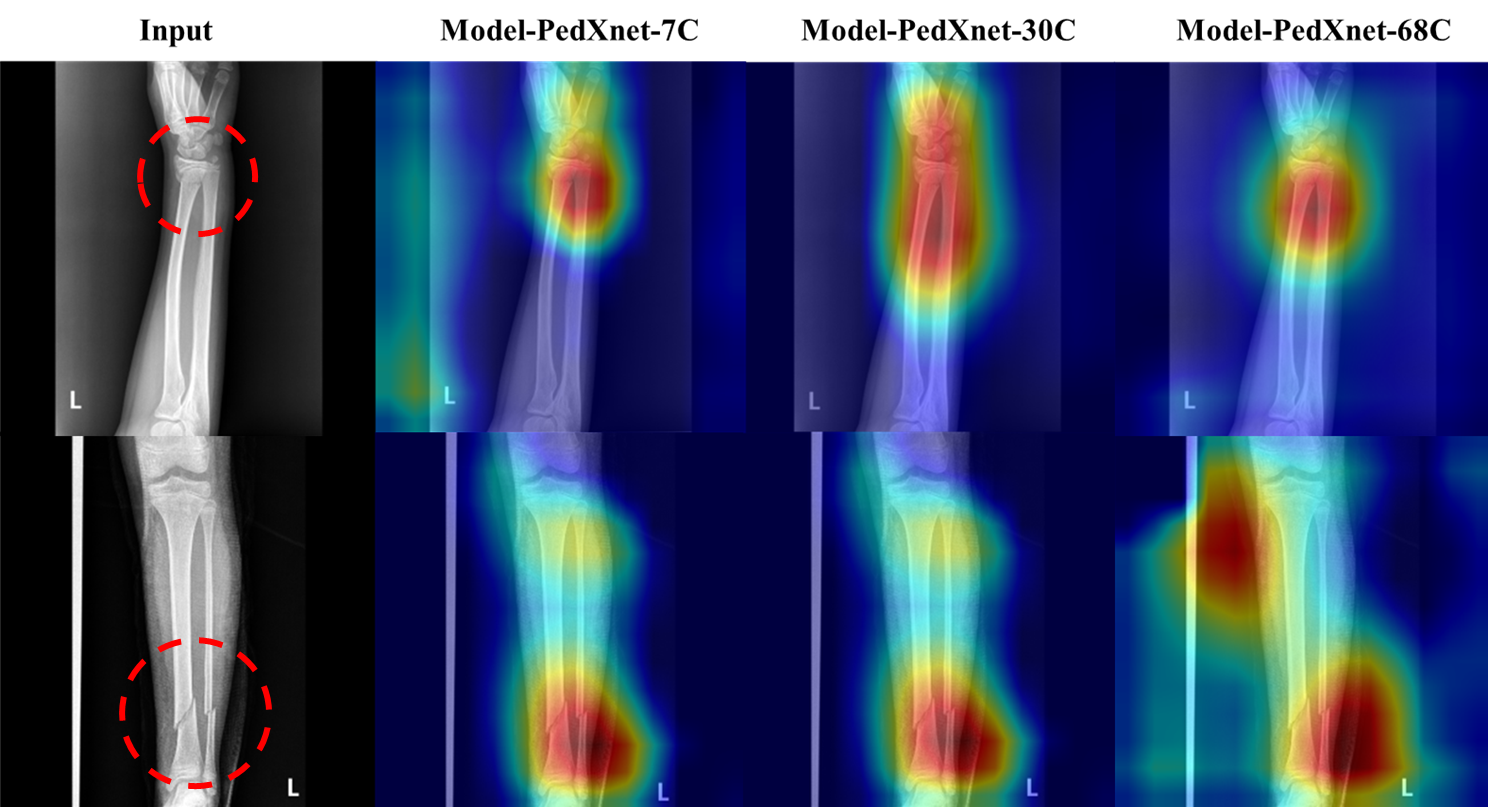


**Supplemental Figure 2.** The plots of Grad-CAM of Model-PedXnet-7C, Model-PedXnet-30C, and Model-PedXnet-68C in the AMC fracture classification task. The left two figures are the case for the lower extremity class and the right two figures are the case for the upper extremity class.

**3. Activation map figures of bone age assessment according to age**

**
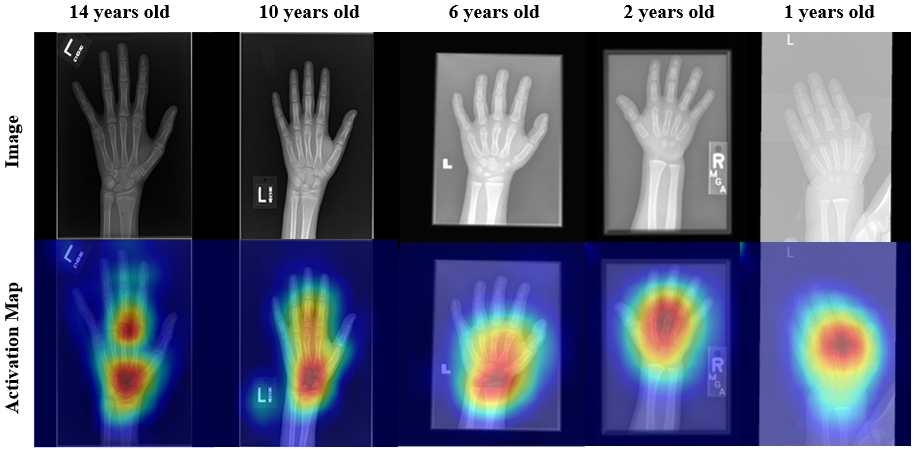
**

**Supplemental Figure 3.** The plots of the activation map of Model-PedXnet-7C in bone age assessment task according to pediatric age. The ages were sorted in descending order from the left. The carpus and metacarpophalangeal joints are critical regions for bone age assessment [39].


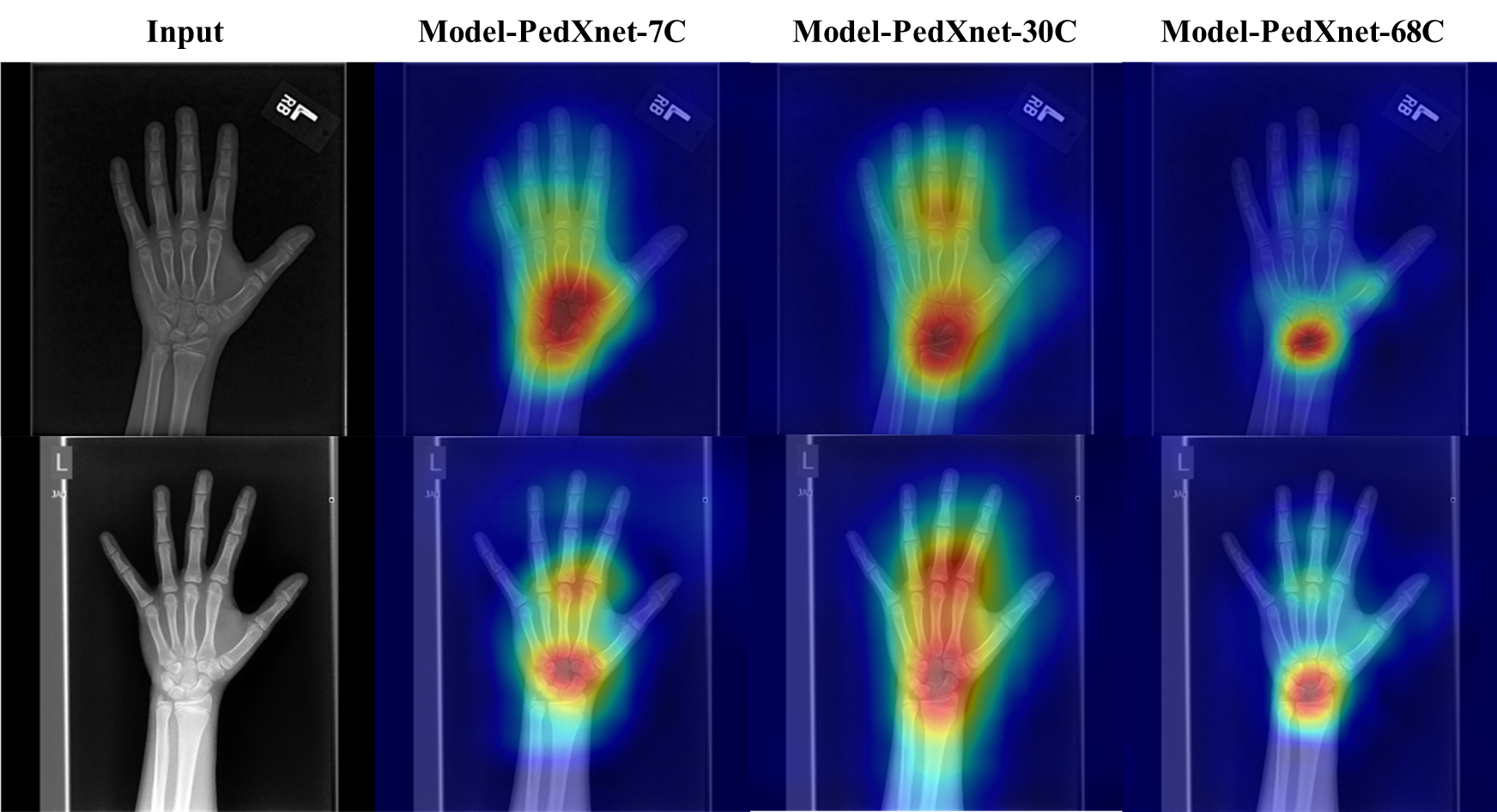


**Supplemental Figure 4.** The plots of the activation map of Model-PedXnet-7C, Model-PedXnet-30C, and Model-PedXnet-68C in bone age assessment task according to pediatric age. The carpus and metacarpophalangeal joints are critical regions for bone age assessment [39].

**4. Expansion of Downstream task**

**AMC Fracture Dataset.** To build a fracture downstream task dataset, we sampled the 1,772 pediatric radiographs from the dataset which was collected over the period of 2018 Jul to 2018 Dec at Asan Medical Center (AMC). Labels were assigned when the keyword "fracture" appeared in the medical report. After that, the presence of fractures in the radiographs was verified through the consensus of two radiologists, each with 12 years of experience. The fracture cases consist of 1,010 radiographs and the normal cases consist of 762 radiographs, and the number of patients is 304. We randomly divided the fracture dataset into training, fine-tuning, and validation sets with an 8:1:1 ratio. The fracture radiographs were confirmed based on the consensus of two radiologists. The fracture downstream task dataset consists of a variety of views of fracture including Ankle, Lower leg, Knee, Femur, Shoulder, Humerus, Elbow, Forearm, and Hand pediatric radiographs. Even though the fracture types were various including artistic fractures and greenstick fractures, we unified all types of fracture to simply solve binary classification.

**Fracture Classification Task.** To solve this fracture classification, the models should extract general features of fracture in the upper and lower extremities of radiographs. We trained the Model-Baseline from scratch and conducted transfer learning using the Model-PedXnet-7C, Model-PedXnet-30C, Model-PedXnet-68C, and Model-ImageNet for the binary classification task of fractures in the upper and lower extremities of radiographs. The classification task loss is defined as the BCE loss. The same preprocessing process and augmentations at the upstream task were performed but the CLAHE was additionally applied to emphasize the bone contrast. For a fair comparison, the same batch size, optimizer, learning rate, and scheduler at the upstream tasks were used except total epoch. The number of epochs at this task is up to 300. However, each model was selected at a converged model that has recorded the highest validation scores. For more information on the baseline characteristics of the AMC Fracture dataset are in the **Supplemental Table 6**.

**Result.** When comparing the Model-PedXnets and Model-Baseline, the AUC scores of Model-PedXNets were remarkably improved in **Supplemental Table 4**. Although Model-ImageNet achieved the highest AUC 0.880 and SEN 0.853 performances, the Model-PedXnet-7C achieved the highest performance with 0.843 in F1, 0.827 in ACC, 0.893 in PPV. We visualized the features of the last InceptionV3 convolution layer of the Model-Baseline, Model-PedXnet-7C, and Model-ImageNet using the Grad-CAM to confirm the representations. The plotted radiographs were randomly sampled in the upper and lower extremities. In **Supplemental Figure** **5**, Grad-CAM shows that the Model-PedXnet-7C focused exactly where the broken areas were in the two radiographs. We added more Grad-CAMs in **Supplemental Figure 1** and **2**. We additionally conducted an ablation study on stress training to apprehend the effects of the pre-trained model more clearly. We only used 30% of the data from the downstream training set. **Supplemental Table 4**, the Model-PedXnets also better performances than the Model-Baseline in a stress training setting. Except for Model-ImageNet's AUC and SPE performances, the Model-PedXnet-30C model achieved the highest overall performance. It is a different result from the highest performances model of Model-PedXnet-7C when using the entire data.

**Supplemental Table 4.** The performance comparisons of the AMC fracture classification task.

| Network | AUC | F1 score | Accuracy | Sensitivity | Specificity | PPV | NPV |
| --- | --- | --- | --- | --- | --- | --- | --- |
| Train 100% of Full Dataset | | | | | | | |
| Model-Baseline | 0.795 | 0.758 | 0.732 | 0.721 | 0.747 | 0.798 | 0.659 |
| Model-PedXnet-7C | 0.877* | **0.843** | **0.827** | 0.798 | 0.867 | **0.893** | 0.756 |
| Model-PedXnet-30C | 0.861* | 0.824 | 0.799 | 0.808 | 0.787 | 0.840 | 0.747 |
| Model-PedXnet-68C | 0.865* | 0.798 | 0.782 | 0.740 | **0.880** | 0.865 | 0.700 |
| Model-ImageNet | **0.880*** | 0.790 | 0.810 | **0.853** | 0.778 | 0.735 | **0.880** |
| Train 30% of Full Dataset (stress training setting) | | | | | | | |
| Model-Baseline | 0.736 | 0.577 | 0.615 | 0.452 | 0.840 | 0.797 | 0.525 |
| Model-PedXnet-7C | 0.783* | 0.760 | 0.732 | **0.731** | 0.733 | 0.792 | 0.663 |
| Model-PedXnet-30C | 0.795* | **0.769** | **0.749** | 0.721 | 0.787 | **0.826** | **0.670** |
| Model-PedXnet-68C | 0.781 | 0.726 | 0.710 | 0.664 | 0.773 | 0.802 | 0.624 |
| Model-ImageNet | **0.805** | 0.747 | 0.732 | 0.683 | **0.800** | 0.824 | 0.645 |

Note: *, p<0.05. DeLong’s test method was adopted for pairwise ROC comparison between the baseline and each model.

**
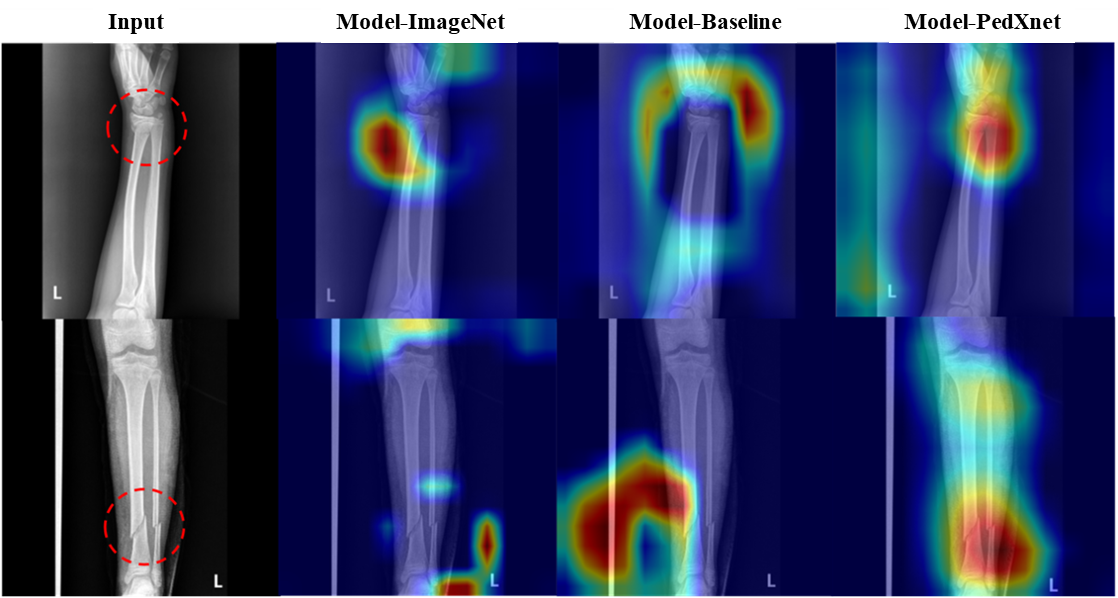
**

**Supplemental Figure 5.** Comparisons of activation maps in the intermediate layer of Model-PedXnet, Model-Baseline, and Model-ImageNet models using Grad-CAM in the fracture downstream task from AMC Fracture dataset.

**5. Statistical Analysis of Datasets for Baseline Characteristics**

Supplemental Table 5. Upstream task datasets baseline characteristics from parsing the DICOM files header.

| Upstream | Model-PedXnet-7C | | Model-PedXnet-30C | | Model-PedXnet-68C | |
| --- | --- | --- | --- | --- | --- | --- |
| Age (mean ± stdev.) | 7.178±5.754 | | 7.601±5.700 | | 7.831±6.066 | |
| Manufacture | GE HealthCare | 39% | GE HealthCare | 40% | GE HealthCare | 37% |
|  | Fujifilm | 25% | Fujifilm | 23% | Fujifilm | 31% |
|  | DK Medical Systems | 10% | DK Medical Systems | 10% | DK Medical Systems | 6% |
|  | Canon | 6% | Canon | 6% | Canon | 7% |
|  | Samsung | 3% | Samsung | 3% | Samsung | 4% |
|  | Etc. | 17% | Etc. | 18% | Etc. | 15% |
| Sex | Male: 50%  Female: 39%  Unknown: 11% | | Male: 52%  Female: 38%  Unknown: 10% | | Male: 51%  Female: 38%  Unknown: 11% | |
| Bit | 14-bit: 63%  12-bit: 20%  10-bit: 17% | | 14-bit: 62%  12-bit: 18%  10-bit: 20% | | 14-bit: 54%  12-bit: 24%  10-bit: 22% | |

Note: The stdev. means standard deviation.

Supplemental Table 6. Downstream task baseline characteristics from parsing the DICOM files header.

| Downstream | AMC Fracture | |
| --- | --- | --- |
| Age (mean ± stdev.) | 7.627±4.991 | |
| Manufacture | GE HealthCare | 2% |
|  | Fujifilm | 4% |
|  | DK Medical Systems | 82% |
|  | Canon | 1% |
|  | Samsung | 6% |
|  | Etc. | 5% |
| Sex | Male: 56%  Female: 38%  Unknown: 6% | |
| Bit | 14-bit: 85%  12-bit: 9%  10-bit: 6% | |

Note: The stdev. means standard deviation.
